# Zinc Binding to Fulvic acids: Assessing the Impact of pH, Metal Concentrations and Chemical Properties of Fulvic Acids on the Mechanism and Stability of Formed Soluble Complexes

**DOI:** 10.3390/molecules25061297

**Published:** 2020-03-12

**Authors:** Patrycja Boguta, Zofia Sokołowska

**Affiliations:** Institute of Agrophysics, Polish Academy of Sciences, Doświadczalna 4, 20-290 Lublin, Poland; z.sokolowska@ipan.lublin.pl

**Keywords:** fulvic acids, zinc soluble complexes, micronutrient organic complexes, metal binding to humic substances

## Abstract

The aim of the study was defined as a complementary analysis of molecular interactions between zinc (Zn) and fulvic acids (FAs) at a broad pH range (3–7), different metal concentrations (0–50 mg dm^−3^) and chemical properties of FAs and their impact on the Zn binding mechanism, stability, and efficiency. The results showed that the complexation reaction prevailed at pH 6 and 7, whereas protons exchange dominated interactions at pH 3. Stability constant of the complexes increased along with pH (logK increased from ~3.8 to 4.2). Complexation was preferred by less-humidified structures of lower molecular mass containing more oxygen groups. The number of fluorophores available for Zn(II) increased from pH 3 to 7 by ~44%. Depending on the pH, complexation involved a bidentate chelate, monodentate and bidentate bridging mode. Zn(II) binding was insufficiently modeled by the classic Stern–Volmer equation and well described by the double logarithmic equation (*R* > 0.94) as well as by a modified Stern–Volmer formula assuming the existence of available and unavailable fluorophore populations (*R* > 0.98). The fluorescence ratio of different fluorophores was proposed as an indicator of the binding affinity of various structures. A positive relationship was found between the fraction of accessible fluorophores and Zn(II) binding at pH 7 determined based on proton release (*R* = 0.91–0.97). The obtained results can find application in controlling the mobility and bioavailability of Zn in different conditions.

## 1. Introduction

Fulvic acids (FAs) belong to one of the most interesting and valuable fractions of humic substances. They are formed by biochemical reactions during the decay and transformation of plant and microbial remains. FAs compared to other fractions of humic substances (humic acids and humins) reveal a lower molecular weight (~500–2000 Da) [1,2], lower sensitivity to pH and better solubility [3], a structure richer in oxygen compounds [2,4] as well as lower aromaticity [5]. FAs find a wide variety of applications, however the greatest potential of these compounds comes from their chelating properties due to a large number of carboxylic, phenolic and alcoholic hydroxyl functional groups (900–1400 cmol kg^−1^) [6,7] as well as others including quinines, semiquinones [8,9], amino and sulphydryl groups [5,10]. As a consequence, FAs exhibit a high sorption capacity to cationic substances, hence they can play a key role in controlling speciation, bioavailability, toxicity and mobility of metals [11,12,13]. According to Bertoli et al. [14], the absence of chelates of metals, like Zn, Cu and Fe, makes these elements insufficient, thus causing deficiencies in the plant and inhibiting root growth. As suggested by Curie and Briat [15], this problem can stem from the predominantly hydrophilic character of cations and the hydrophobic nature of pores or openings in roots and leaves. The chelating effect of FAs can reduce the cation-positive charge and facilitate the migration of micronutrients to the plant. On the other hand, the lack of knowledge on interactions and complexing properties of FAs or other fractions of soil organic matter can pose a risk of heavy metal migration [16,17] and stabilize toxic species in drinking water [18,19]. Translocation of heavy metals to plants can increase the risk of food-chain contamination [20].

Zinc (Zn) belongs to essential micronutrients needed for the proper growth of plants. However, both high accumulation and insufficiency of this metal can be harmful [21,22,23]. An especially relevant problem is related to Zn deficiency caused by high-yielding plants, crop intensification and continuous removal of Zn native to soil [12]. To maintain the optimal amount of this element in a bioavailable form is challenging because it mainly forms difficult-to-resolve phosphates, carbonates, sulfides or compounds with Fe or Mn hydroxides [24]. In turn, free Zn ions, more typical of acidic pHs, are characterized by high mobility and are easily leached from soil [25].

The interaction of FAs with Zn can have an enormous potential for the agricultural sector in the development of efficient preparations of stable, highly bioavailable compounds supplementing both organic carbon and zinc [12]. Some studies conducted on Zn interactions with natural organic matter, dissolved organic matter or humic acids have indicated a possible increase in bioavailability of Zn in such connections. In their studies of heavy metals interactions, He et al. [21] noticed a decreasing concentration of free Zn(II) ions in the presence of dissolved organic carbon, which could prove Zn binding. Güngör and Bekbölet [6] showed a mobilizing effect of FAs and humic acids on Zn contained in soil. The studies held by Zhang et al. [26] on interactions of Zn with dissolved organic carbon at pH 3–9 demonstrated that speciation, bioavailability and toxicity are determined by dissolved organic carbon with the key participation of oxygen-containing groups and the proton replacement process. Experiments conducted by Boguta et al. on interactions of humic acids with Zn indicate a high importance of pH, metal concentration and the chemical properties of organic molecules on the stability of formed complexes [27,28].

Zn and FAs connections seem promising due to the favorable properties of FAs. However, the mechanism of these processes is still poorly understood, and the selection of appropriate conditions for obtaining compounds of the highest stability and microelement content causes multiple problems and requires further comprehensive research. The main difficulty is that both FAs molecules and Zn(II) ions show high sensitivity and variability to changes in pH, ionic strength, free metal ion concentration and the presence of competing ions [10,11]. Zn at low pH and in the absence of organic ligands is present as hydrated Zn^2+^_(aq)_ [29]. However, various hydroxide species of different charge can be formed from neutral to alkaline pHs [30]. The pH also affects the size, optical properties, molecular orbitals and sphero-colloidal configuration of organic molecules [31,32,33]. The complexity of interactions with Zn is also influenced by the properties of FAs originating from various sources. Soil, animal, vegetal or microbiological FAs can exhibit a different chemical composition and conformation revealing a varied molecular weight, humification degree, content of O, C, H and N, functional groups, aromatic and aliphatic structures [14,34,35]. In their studies on complexes between humic substances and ions Cu^2+^, Fe^2+^ and Zn^2+^, Santos et al. [4] noted that the interactions were influenced by the origin and structure of the organic samples used and should be evaluated separately. Moreover, high FAs heterogeneity and the variability of environmental factors may result in a high diversity of possible types of interactions, including ion-exchange, surface-adsorption, coagulation, peptization, chelation, metal bridging and hydrogen bonds participation [14,17,27,36].

There is not much research to be found in the literature on the subject offering a comprehensive description of binding mechanisms in the FA-Zn system and considering the influence of various factors and chemical properties of FAs. The complicated nature of FAs causes this field to remain largely unexplored, and the existing knowledge is still insufficient. Therefore, the main goal of our studies was the analysis of interaction mechanisms between FAs and Zn(II), including the effect of: (1) pH, (2) Zn(II) concentration, and (3) properties of FAs derived from chemically different soils. We also performed the analysis of relationships between the parameters describing Zn(II) binding. Such comprehensive studies will enable the control of interaction efficiency in the FAs-Zn(II) system as well as enabling the development of natural and economically efficient preparations with high fertilization efficiency.

## 2. Results and Discussion

### 2.1. Fulvic Acids

FAs isolated from various soils differed significantly in terms of chemical and structural properties (Table 1). The H/C atomic ratio showed the highest value for FA2 (0.94) while indicating the lowest degree of condensation of aromatic rings, and thus the greatest proportion of aliphatic structures in relation to FA3, which showed the lowest H/C value (0.85) [13,37]. The highest O/C and O/H ratios were determined for FA2 sample, while the lowest ones for FA3, which indicated a higher content of oxygen structures and as a higher humification degree of FA2 as compared to FA3. The degree of internal oxidation, ω, as in the case of the O/C and O/H ratio, was the highest for FA2 and the lowest for FA4 and demonstrated the binding of oxygen to both carbon, hydrogen and nitrogen.

The total content of COOH and OH functional groups was reported previously [35]. It was the highest for the FA1 and FA4 structures, which, according to the authors suggested high sorption capabilities of the samples. The number of OH groups was about 2–3 times smaller than the number of COOH groups. The total of COOH and OH groups in FAs was significantly higher than in the case of humic acids isolated from the same soils [35], which suggests a potentially greater significance of the FA fraction in the agricultural context of interactions with metals. Interestingly, the higher number of COOH and OH groups for FA4 do not correspond with the highest value of the O/C and O/H ratio. This suggests that FA2 with the highest value of O/C and O/H also contained oxygen structures other than COOH and OH.

The results of UV-Vis spectroscopy showed that the lowest and highest Q465/Q665 values were attributed to FA2 and FA4, respectively, indicating a greater conjugation of double bonds, the highest humification degree and molecular weight for FA2, and the weakest humification stage and structural units of lower molecular weight for FA4 [38]. The Q280 value was similar for all samples, suggesting slight differences in FAs aromaticity [39]. The Q254/Q436 parameter was the highest for FA1, meaning that it contained the highest number of UV absorbing functional groups compared to the colored ones [40]. The ash content in the FAs samples ranged from 0.63% to 1.63%, which proves that the mineral parts had been washed properly and proves the high quality of obtained preparations for further experiments related to interactions with Zn(II) ions.

### 2.2. Fluorescence Properties of FAs

**Figure 1:**
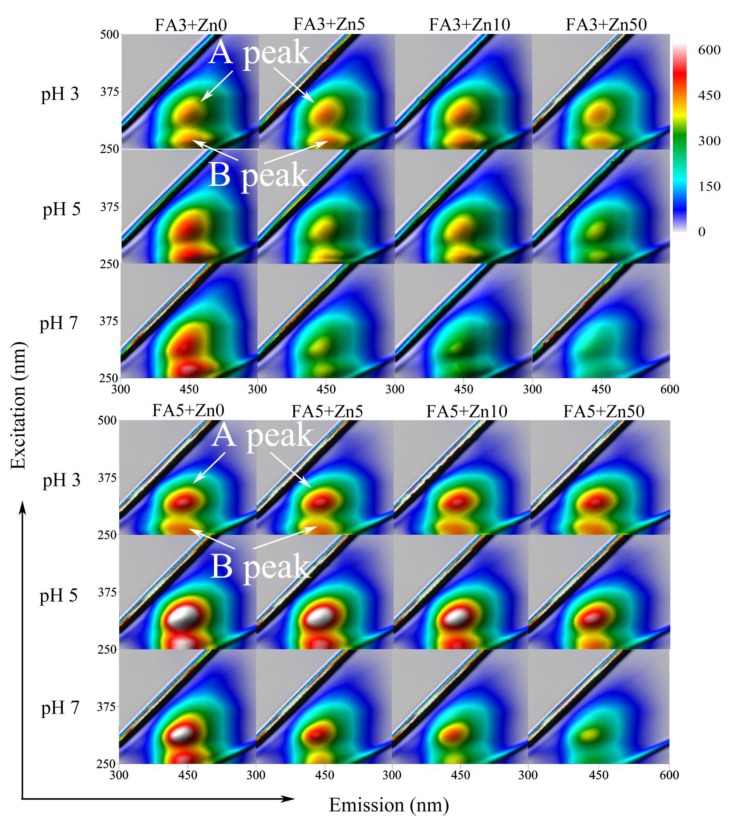
Excitation-emission matrices (EEM) for FAs and FAs-Zn(II) solutions as a function of increasing pH and Zn concentrations.

The results of the fluorescent analysis of FAs without Zn(II) revealed the presence of two spectral regions (peaks marked as A and B). The exemplary excitation-emission matrices (EEM) of FAs at different pH are shown in Figure 1.

The maxima of fluorescence intensities (FI) for the A-peak were located at ex/em: ~320 nm/~440 nm and were attributed to the humic substances of higher molecular weight [41] and Vis fulvic-like systems [42]. Fluorescent regions, located at longer excitation wavelengths, can be also related to the greater number of conjugated aromatic π-electron structures with electron withdrawing functional groups such as C=O [43,44]. The maximum of the A-peak was slightly shifted (5–10 nm) toward the longer wavelengths as the pH decreased from 7 to 3, which could be associated with a conformational change in the structure of FAs. B-peak was observed at ex/em of ~260 nm/~440 nm, and this position remained unchanged under the pH conditions. Additionally, for FA3 from black soil, the B-peak was located at longer wavelengths and showed the maximum at ex/em: ~270 nm/~450 nm. Fluorophores of the B-region are related to humic substances of lower molecular weight [41], UV fulvic-like systems [42], simple structural components of wide heterogeneity, low- molecular weight and aromatic polycondensation [27]. Considerable differences between the studied FAs were observed in maxima intensities, which is shown in Table 2.

The highest intensities of A- and B-peaks were reported for FA1, whereas the lowest for FA3, independently on pH. This indicates the highest number of bearing electron-donating substituents e.g., hydroxyl, metoxyl and amino groups in FA1 [45,46] and the highest number of bearing electron-withdrawing substituents, such as carbonyl and carboxyl groups, in FA3 [47]. For most of the studied FAs, A fluorophores exhibited a higher fluorescence intensity as compared to the B ones. The exception was FA3 which was characterized by peak A and B of a similar fluorescence signal. The increase of the pH from 3 to 7 caused an increase in FI of most FAs (except for FA5 with the maximum at pH 5). According to Hudson and Reynolds [48] and Patel-Sorrentino et al. [49], such changes are a consequence of structural transitions in the molecule consisting in assuming a coiled, spherocolloidal shape at low pH and a gradual expansion to linear form at increased pH. Long-tailed configuration of humic substances at neutral and alkaline pHs, resulting from repulsion between negatively charged function groups [27], makes fluorophores more exposed and reveals their high fluorescence intensity [50]. Protonation of functional groups at acidic pH leads to polymerization and aggregation of molecules, as well as to a stronger hydrogen bonding effect and, in consequence, to a sharp decrease in fluorescence intensity.

### 2.3. Fluorescent Quenching Titration in the Analysis of Zn(II) Binding Properties of FAs

**Figure 2:**
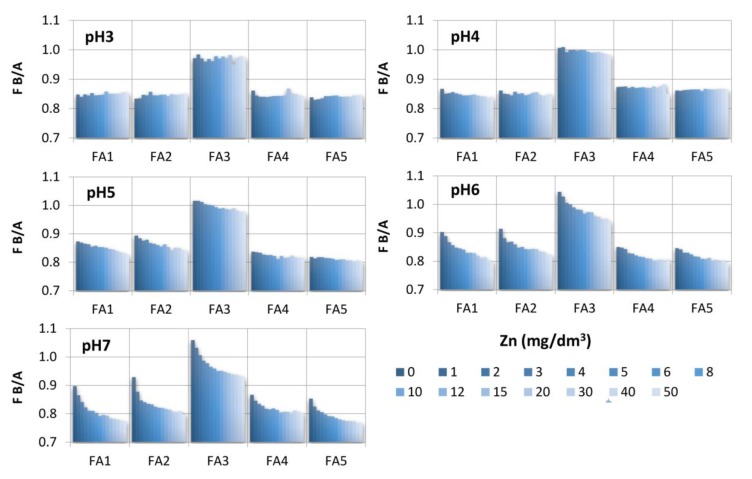
Differences in the quenching of A and B peaks expressed as changes in ratio of B and A intensities as a function of increasing Zn(II) concentration.

Previous studies show that the process of complexation of organic compounds with metal ions can also be observed in the form of static quenching due to the quenching nature of metals [51,52]. The results of our studies demonstrate that the supplementation of Zn(II) ions caused FI quenching of FAs, both in the A- and B-region. Visualization of this process for FAs with a rising Zn(II) concentration is provided on EEM spectra in Figure 1. The most intensive quenching occurred in samples at high pH, whereas changes observed at pH 4 and, especially, at pH 3 were infinitesimal. Low quenching at acidic conditions can be attributed to the high level of competition between hydrogen and the metal for the negatively charged active sites [27,41]. Intensive quenching at pH 7 was due to better access of the metal to functional groups [53]. The FI decrease under influence of Zn(II) was different for A- and B-areas of the studied FAs, which was the most visible for the top pH systems. These inequalities are shown in Figure 2 as changes of the ratio of B and A intensities in relation to increasing Zn(II) concentration. The results show that the addition of Zn(II) caused a gradual decrease in the B/A ratio, indicating a stronger decrease of FI in the B region as compared to the A one. A particularly strong reduction in the B/A ratio was observed for FA2 (already at the initial Zn concentration). This data suggests differences in affinity of the studied fluorophore groups for Zn(II) ions. Chemical heterogeneity of the binding sites in the FA structure was also reported in the studies of Wang et al. [10] conducted on Pb(II) interactions as well as in the experiments of Song et al. [11] related to the proton binding properties of FAs sub-fractions. Our results demonstrate that A fluorophores, i.e., conjugated aromatic π-electron structures of higher molecular mass, exhibit lower affinity for Zn(II) ions compared to the structures of the B region, i.e., simpler structural components of wide heterogeneity and lower molecular weight. It should be noted that the FA2 sample was characterized by the highest values of ω, O/C, O/H and H/C ratio attributed to oxygen-rich and low-humified structures, so it can be concluded that such functionalities can also be identified with B fluorophores. Moreover, oxygen-containing groups are sensitive to pH changes and reveal the highest surface negative charge at high pH [54,55]. This can be a reason for the most profound changes of the B/A ratio at pH 7.

**Figure 3:**
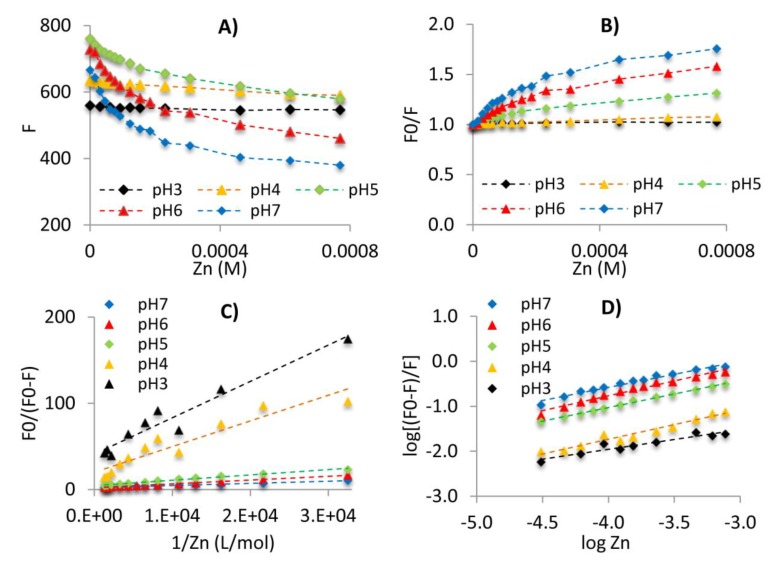
The analysis of fluorescence quenching data for A-peak of the FA5-Zn(II) system at pH 3–7. (**A**) fluorescence quenching profiles with Zn(II) increase; (**B**) Stern–Volmer plots; (**C**) modified Stern–Volmer plots; (**D**) double logarithmic plots.

Fluorescence quenching was the strongest at the initial doses of Zn(II) and weakened with increasing metal concentration to reach the plateau at higher levels of Zn(II) and indicating saturation of the accessible sites. However, quenching was not complete in any of the tested systems, despite the excess of metal, which is shown in Figure 3a. In conclusion: (1) some fluorophores do not reveal affinity for binding Zn(II) ions, or/and (2) the binding of Zn(II) ions in one place decreases the binding efficiency in the neighboring place, or/and (3) spatial conformation of the molecule hindered access to metal ions. The last conclusion seems to be confirmed by the plots in Figure 3a showing that the degree of FI quenching with Zn(II) ions increases with the increasing pH, i.e., along with the degree of spatial development of the molecule. Studies of Sahu and Banerjee [56] related to the complexes of FAs and humic acids with Cu, Cd and Pb reveal that low metal concentration leads to the formation of stronger complexes due to the preferential binding to functional groups of stronger binding strengths. According to these authors, higher metal concentration occupied weaker functional groups, thus forming weaker complexes.

For systems with higher pH (5–7), the increase in Zn(II) concentration caused a slight shift of the maxima of A and B areas towards shorter emission waves (shift ~5 nm). Li et al. [57] observed a similar shift for Cu(II), Pb(II) and Cd(II) binding to FAs by fluorophores at Ex/Em 390 nm/485 nm. They attributed it to a decrease of aromatic rings, reduction of conjugated bonds or conversion of the linear ring system to a non-linear one. In the studies of Hg(II) complexation by dissolved organic matter, Wu et al. [58] observed an increase in the emission and excitation wavelength during the first 20 s of interaction. According to the authors, such changes may indicate the occurrence of structural transitions of organic molecules containing conformational changes or changes to rigidness. As regards our results, changes occurred at higher pH where the structure was more flexible and mobile than at acidic pH. Therefore, changes to the rigidness and conformation of molecules might be one of the drivers in mechanism of interaction with Zn(II) ions.

### 2.4. Quantification of the FA-Zn(II) Binding Process

The Stern–Volmer equation was used for a quantitative description of fluorescence quenching in the FA-Zn(II) system:

(1) $$\frac{F_{0}}{F}=1+k_{q}\tau_{0}\left[\mathrm{Zn}\left(\mathrm{II}\right)\right]=1+K_{SV}\left[\mathrm{Zn}\left(\mathrm{II}\right)\right],$$

where *F*_0_ and *F* were the FI value for FAs solutions with Zn(II) absent and present, respectively, *k_q_*—rate of energy transfer, *τ*_0_—fluorescence lifetime, [Zn(II)]—metal concentration, *K_Sv_*—Stern–Volmer quenching constant—related to the quencher ability to quench the FI, regardless of the mechanism applied. Some exemplary fluorescent data elaborated using the Stern–Volmer equation are shown in Figure 3b.

An attempt to determine *K_SV_* from the slope of *F*_0_/*F* vs. [Zn(II)] plots showed that graphs obtained for all the studied systems deviated from the straight-line course, which reveals a rather concave-down response (Figure 3b). Such a trend may indicate complex quenching due to the presence of the population of fluorophores available and unavailable for complexing [1]. The equation parameters should not be determined in this case, due to a high probability of overestimation of results. Cases of occurrence of the fraction of accessible and inaccessible fluorophores in interactions of humic substances and metals had been reported previously for other cations. Studies done by Fu et al. [59] revealed the presence of inaccessible fluorophores in the Hg(II) binding by humic substances. Concave-down courses for the Stern–Volmer equation were also noted by Esteves Da Silva et al. [60] for the systems of FAs with Cu(II), Fe(III) and UO_2_^2+^ as well as by Chen et al. [61] for the binding of Cu(II) by dissolved organic matter. Zhao and Nelson [1] reported a concave-down response in the interactions of FAs with Tb(III) and Pd(II), a linear course for Cu(II) binding and a concave-up response for FA-Fe(III). The facts above, as well as the results obtained in our studies for Zn(II) ions binding, indicate that the mechanism of fluorescence quenching largely depends on type of metal as well as on the structure of fluorophores, and varies for humic substances, humic acids, FAs and dissolved organic matter. The existence of fluorophore population inaccessible to metal may be due to the fact that they are not accessible for complexation or do not have complexing properties. Hays et al. [62] suggest that the nonlinear response in FA and metal interactions can also be linked to the overlapping of different kinds of fluorophores and the shielding of binding sites or fluorescence. Due to the nonlinear course of Stern–Volmer plots for FA-Zn(II) solutions, the modified Stern–Volmer equation was therefore used in our studies to illustrate fluorescence quenching that makes allowances for both the accessible and inaccessible population of fluorophores [11,59,63]:

(2) $$F_{0}=F_{0a}+F_{0b},$$

where, *F*_0*a*_ was assigned to the fraction of fluorophores accessible to Zn(II) ions, and *F*_0*b*_ was related to inaccessible fluorophore moieties. The changes of the fluorescence signal under the influence of metal can be expressed in this case as:

(3) $$F=\frac{F_{0a}}{1+K_{a}\left[Zn\left(II\right)\right]}+F_{0b},$$

where, *K_a_* is the Stern–Volmer constant or conditional stability constant related only to accessible fluorophores. The fraction of fluorophores available for Zn(II) can be introduced as *f_a_*:

(4) $$f_{a}=\;\frac{F_{0a}}{F_{0b}+F_{0a}},$$

In this case, Equation (3) is given by:

(5) $$\frac{F_{0}}{F_{0}-F}=\frac{1}{f_{a}K_{a}\left[Zn\left(II\right)\right]}+\frac{1}{f_{a}},$$

**Figure 4:**
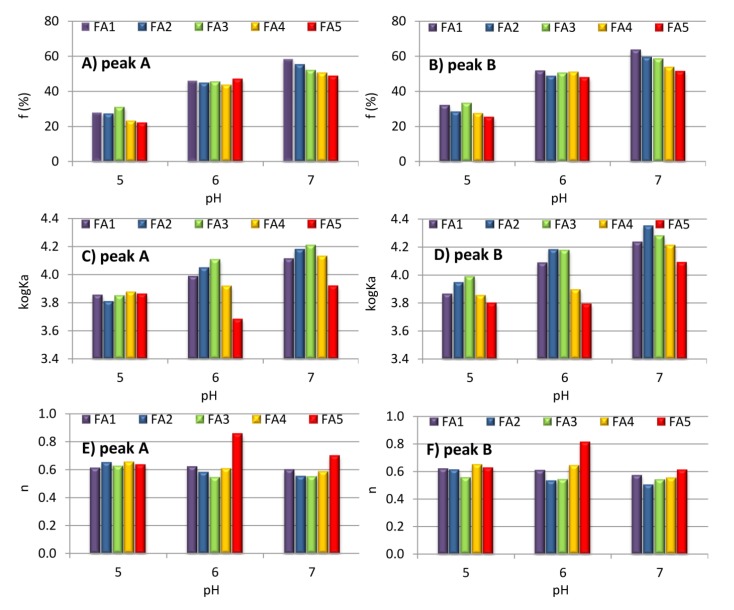
Changes of the binding parameters of FA-Zn(II) complexes as a function of the pH for A and B peaks: (**A**,**B**) fraction of the accessible fluorophores; (**C**,**D**) conditional stability constant; (**E**,**F**) number of binding sites.

*K_a_* and *f_a_* can be determined from the slope and intercept of *F*_0_/(*F*_0_ − *F*) versus 1/[Zn(II)]. Plots illustrating how the modified equation works at pH 3–7 were shown in Figure 3c. The obtained characteristics reveal high linearity for the FA-Zn(II) systems at pH 5–7 (*R* > 0.97), which permits to determine the *K_a_* and *f_a_* values for these experiment sets (Table 3). For the majority of the systems at pH 3 and 4, the correlation coefficients were too low to determine binding parameters. This can be attributed to slight quenching of fluorescence by metal ions due to strong competition with protons. Under acidic conditions, the micellar structure of FAs also significantly increased the population of fluorophores unavailable in the complexation process. Changes to *K_a_* and *f_a_* as a function of pH for the A and B fluorescent regions are shown in Figure 4a–d. The *K_a_* values were slightly higher for B-peaks and increased with the pH, hence indicating the highest stability of the complexes formed at pH 7. The increase in the stability of FA-Zn(II) compounds with a simultaneous increase in the pH may result from higher spatial availability of metal to structures such as carboxyl and phenol groups responsible for the formation of strong complex bonds. In their studies on interactions between humic acids and antibiotics, Wang et al. [50] reported an increase of stability constant with the pH up to pH 8. These results revealed that complexation may be hampered at acidic pH due to interactions of the hydrophobic surface of humic acids (protonated, aggregated structures) with hydrophilic quencher, as well as that the binding was stronger at neutral and alkaline pH due to interactions of the hydrophilic surfaces of humic acids with hydrophilic quencher. Sahu and Banerjee [56] reported that the conditional stability of the complexes of humic and fulvic acids with Cu, Cd and Pb rose with an increase in the pH due to the higher potential of ionized groups to form strong complexes. This is also consistent with the arguments of Piana et al. [64] on the increased stability of metal complexes at the pH ranging from 4 to 8 due to higher overall negative surface charge, originating not only from carboxylic groups but also from phenolic groups, to which more cationic species can bind. According to Garcia-Mina [65], binding sites, consisting of both carboxylates and phenolic groups, can be involved in the formation of stronger types of complexes.

Higher values of stability constants for fluorophores from the B area indicated a greater stability of connections of Zn ions with the structures of lower molecular weight, which is typical of FAs. These results correspond to the ones obtained by Wei et al. [66]. They reported that stability constants of Cu(II) and Eu(III) binding by water extractable organic matter revealed higher values for the component typical for FAs than for the component typical of humic acids. The authors suggested that these differences could be attributed to a greater number of carboxyl and phenolic groups in FAs. On the other hand, Enev et al. [63], in the binding studies of Hg, Cu, and Pb at pH 3.7–5.15, determined higher stability constants of humic acids-metal complexes as compared to FAs complexes and justified the results by a high content of acidic functional groups and aromatic structural units forming highly stable, bidentate complexes with metal. Different results obtained for different metals, different structures of organic molecules and various conditions lead to a conclusion that the stability of connections of humic substances with metals is strongly dependent on the conformation and composition of ligands, pH, and type of cation.

Population of the fluorophores (*f_a_*) accessible to Zn(II) increased along with the pH and was slightly higher for the B region and the highest for FA1. An increase in the pH to the neutral or alkaline values results in the dissociation of further functional groups, including also phenolic structures, which can raise *f_a_* values. Possible participation of phenolic groups of humic substances in metal complexation was previously suggested by Gungor and Bekbolet [6] or Santos et al. [4]. Moreover, in their studies on the complexes of humic substances and metals, Garcia-Mina [65] proposed two-binding patterns: one involving the acidic range of pH with protonated amino groups and carboxylates, and the other alkali conditions involving phenolic groups and carboxylates. In our studies, a pH increase caused an increase in negative surface charge and, in consequence, the repulsion of individual structures to protect the molecule against aggregation [27] and, thus, to highlight subsequent fluorophores available for interactions with Zn(II). Furthermore, FA1, which reveals the highest *f_a_* value, was also characterized by the largest number of COOH groups, which confirmed the role of these structures in the B fluorescent region and in Zn(II) binding. For an extra analysis of interactions between FAs and Zn(II), a double logarithmic equation was used:

(6) $$log\frac{F_{0}-F}{F}=logK_{b}+nlog\left[Zn\left(II\right)\right],$$

based on which binding constant (*K_b_*) as well as the number of binding sites (n) were determined. The log-log plots (Figure 3d) exhibited high linearity (*R* > 0.94), indicating good fit of the model with experimental data. The obtained results show that the n value was similar for A and B peaks and did not exceed 1, which suggests that more binding sites of FAs were required to form a complex compound with Zn(II). A similar mechanism of interaction was also described in the studies of Fu et al. [59] related to Hg(II) complexation by heated and unheated humic substances for which n values ranged from 0.39 to 0.47. In our studies, the n and *K_b_* values generally increased with the pH, however a slight decrease of these parameters was observed for some samples at pH 7, probably due to the formation of hydrolyzed ZnOH^+^ species [67]. Generally, *K_b_* values were lower than *K_a_* ones, probably due to the macroscopic meaning of *K_b_*. However, it should be noted that use of the log-log equation is disputable for the interaction of metals with fulvic acids. The equation was originally proposed to describe protein complexation, and one of its basic assumptions was the slope of the log-log plot greater than 1, which meant “positive cooperative binding” taking place when the binding at one site enhanced the binding at subsequent sites [56].

### 2.5. FTIR Spectra of FA-Zn(II) Compounds

**Figure 5:**
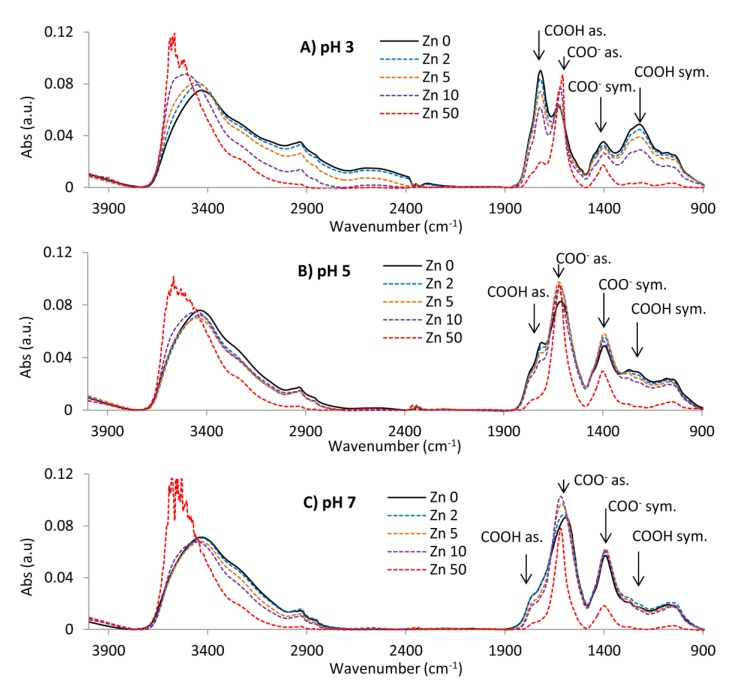
FTIR spectra of the FA4-Zn(II) complexes at increasing metal concentration and at (**A**) pH 3; (**B**) pH 5; and (**C**) pH 7.

The FTIR spectra of FAs without Zn(II) revealed the presence of absorption bands typical of this fraction of humic substances. Slight differences between FAs from different soils concerned mainly the intensity of individual bands, and to a lesser extent—wavenumber shifts. Some exemplary FTIR characteristics are shown in Figure 5 (black, solid lines). Some of the observed signals can be considered important in terms of interaction with Zn(II): (a) wide intensive band at 3435 cm^−1^ attributed to the N-H stretch of amines and O–H of hydroxyl groups in hydrogen bonds, carboxylic acids, alcohols and phenols [5,13,16], (b) vibrations at ~1710 cm^−1^ related to C=O stretching of protonated carboxyls, quinines, cyclic and acyclic aldehydes and ketones [42,68], (c) band at ~1625 cm^−1^ refers to C=O bonds of deprotonated COO-, C–O and C-N stretching in amides [13,69] as well as vibrations of –C = C– on benzene rings [16,42], (d) peak at ~1400 cm^−1^ reflecting aliphatic –C-H deformation, -COO- symmetric stretching and -O-H deformation [5], (e) vibrations at ~1220 cm^−1^ corresponded to C–O stretching in phenols, carboxylic acids and ethers [68,70] and (f) band at ~1070 cm^−1^, indicating the presence of polysaccharides, ethers and alcohols attributed to the stretches of C–O bond [5,13]. The increase of pH caused a gradual reduction of the bands at ~1710 cm^−1^ and at ~1220 cm^−1^ and enhancement of the bands at ~1625 and at ~1400 cm^−1^, which was associated with the successive deprotonation of surface COOH groups and, consequently, an increase of the COO^−^ fraction.

The addition of Zn(II) ions caused a decrease in the absorption bands at ~1710 cm^−1^ and at 1220 cm^−1^ indicating the binding of cations by COOH groups (Figure 5). This mechanism was also confirmed by the increase in absorption bands at ~1625 cm^−1^ and at ~1400 cm^−1^ typical of deprotonated COO^−^ groups. According to Ahmed et al. [71], changes in the position and intensity of bands around 1700 and 1600 cm^−1^ result from coordinate bond formation between lone electron pairs on oxygen atoms and empty d-orbitals in heavy metal. Band usefulness at ~1710 cm^−1^ for the observation of metal complexing processes by humic substances was also demonstrated, among others, by Jerzykiewicz [72] in the studies of mercury complexation and by Li et al. [57] during the investigation of the role of functional groups in heavy metals binding by FAs in lake sediments. In our studies, absorption reduction typical of COOH groups was the most intense at pH 3 and the weakest at pH 7, indicating the most intense Zn(II) exchange with protons at pH 3 and the weakest one at pH 7. It can be explained by the fact that most COOH groups at pH 3 are protonated and bands at 1710 cm^−1^ and 1220 cm^−1^ were strong while revealing a high potential for significant reduction under Zn(II) influence. In turn, most COOH groups at pH 7 were already dissociated the revealed a low intensity of these bands and, consequently, not so spectacular absorption weakening after Zn(II) supplementation. Interestingly, fluorescent studies (paragraph 2.4) prove the highest binding of Zn(II) ions at pH 7. It could be explained by the fact that complexation may also take place through dissociated ligands as well as other donor atoms, e.g., nitrogen-containing structures. In the studies on binding Cu, Pb and Cd by FAs obtained from lake sediments, Li et al. [57] classified phenolic and carboxyl groups as weaker binding sites as compared to nitrogen-containing groups. They also showed an increase of adsorption capacity of Cu, Pb and Cd ions with the higher pH from 3 to 6 due to deprotonation of functional groups, facilitating the electrostatic attraction of metal.

Therefore, it should be concluded that the exchange of Zn(II) ions with protons is the only possible reaction mechanism, typical of low pH values. Given the above, an increased number of bound Zn(II) at the higher pH results to a lesser extent from exchanges with protons and more from attraction and complexation of positive cations to negatively charged functional groups, as well as from a more linearly developed structure that facilitates the penetration of zinc cations. These assumptions seem to be confirmed by the studies of Saab et al. [73] on pH effect in aquatic FAs. It shows that electrostatic interactions are weak for pH 3, and hydrogen bonding is responsible for the formation of aggregates while at pH 9 the electrostatic interactions are strong due to ionization of phenolic groups and low hydrogen interaction. In our studies, aggregation at pH 3 caused an additionally steric effect which hindered access of metal ions. It was well visible as not completely reduced band at ~1710 cm^−1^ (even at the highest Zn concentration). The absorption band of OH group at ~3435 cm^−1^ changed its location towards longer wavelengths after Zn(II) addition (blue shift), indicating a change in the coordination sphere of the complex [27] and involvement of oxygen from phenolic groups to complex formation [74]. The blue shift was also observed for band at ~1625 cm^−1^ for complexes at high pHs indicating a complex formation with a higher covalent nature [74].

**Figure 6:**
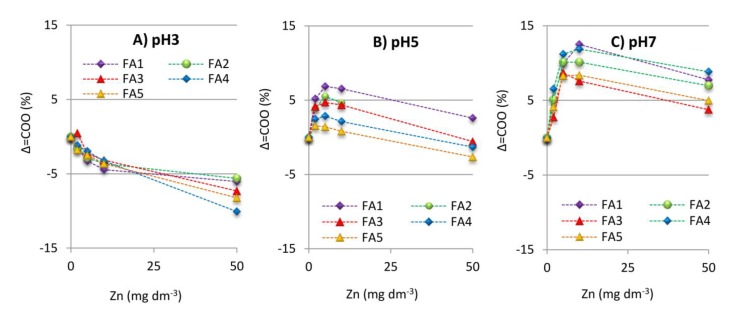
The distance between asymmetric and symmetric COO^−^ bands (∆COO^−^) of the FA-Zn(II) compound in relation to the distance for ionic form of FAs for the systems at (**A**) pH 3; (**B**) pH 5; (**C**) pH 7.

The mechanism of interaction between Zn(II) and FAs can be additionally inferred from analyzing the wavenumber difference between asymmetric and symmetric COO^−^ bands (∆COO^−^) of the FA-Zn(II) compound in relation to a similar distance for the ionic form of FAs [71,75,76]. The ∆COO^−^ parameter (band separation value) is positively related to the mode of coordination in the sequence: monodentate > uncomplexed carboxylate ion(ionic) = bidentate bridging complexes > bidentate chelate complexes. Changes in this distance presented as a function of Zn(II) concentration are depicted for the studied compounds in Figure 6. A decrease in the value of ∆COO^−^ is observed at pH 3, which suggests the bidentate chelate mode of Zn(II) binding (the metal ion interacts equally with the two oxygen atoms of a COO^−^). Such mechanism can be attributed to the spatial shape of particles at acidic conditions. Greater aggregation of the structure means that the functional groups reduce distance to each other and that cations can be bound more easily by two oxygen atoms.

The metal binding mechanism is more complex for the studied samples at the higher pH: the monodentate mode is predominant at low Zn(II) concentrations (the metal ion interacts with only one oxygen atom of a COO^−^ group) while the bidentate bridging mode begins to be more relevant above 5 mg dm^−3^ of Zn(II) (the metal ion binds to one of the two oxygens in a COO^−^ group and another metal ion to the other oxygen atom). The observed trends applied to all tested FAs, which suggests the same reaction mechanisms regardless of FAs properties. The factor differentiating the coordination mechanism was the pH (Figure 6a–c) due to varied dissociation of the functional groups and changes in the spatial shape. Similar results were obtained before at pH 7 for the interactions of Zn(II) ions with humic acids containing a high number of O-functional groups [27]. In those studies, however, humic acids at pH 5 revealed a clear change in the complexation mechanism as compared to the interaction at pH 7, which is not so well pronounced in our current studies on the FAs fraction at pH 5 and 7. This is in line with the observation of Tipping [77] that smaller FAs have fewer opportunities to undergo a conformational change.

Among the studied FAs, the largest differences in interaction mechanisms at various pHs were found for FA4, probably due to the highest content of carboxyl and acidic hydroxyl groups which are strongly sensitive to pH changes. This also indicates significance of the above structures in Zn(II) complexation. Alloway et al. [78] as well as Zhang et al. [79] report that stable multidentate and multinuclear chelate sites are formed from the mix of these groups or by N- and S- structures.

### 2.6. Zinc-Protons Competitive Interactions

**Figure 7:**
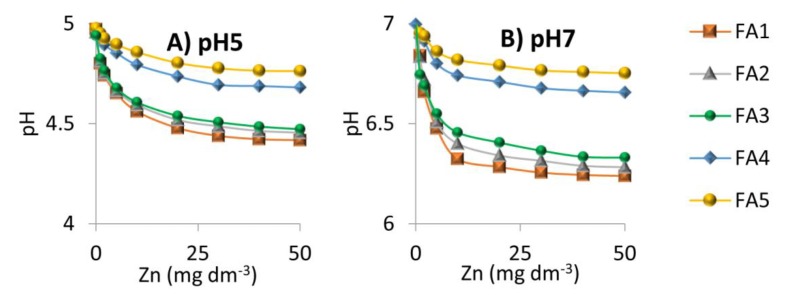
The pH drop of the FAs solutions during interactions with increasing Zn(II) concentration for the systems at (**A**) pH 5; (**B**) pH 7.

The results of potentiometric titration prove that one of the mechanisms governing the interaction in FAs-Zn(II) system was the exchange of protons with metal ions. Addition of Zn(II) ions reduced the pH of FAs solutions, indicating that metal binding and proton release into the solution took place. The dynamics of pH decrease was the highest at low Zn(II) concentrations and weakened along with increasing the metal dose until changes became insignificant (Figure 7).

**Figure 8:**
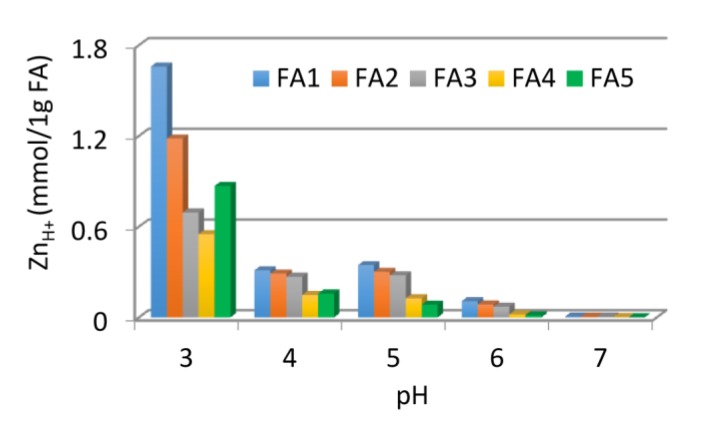
Efficiency of Zn(II) binding by FAs during exchange with protons as a function of pH.

The reason for this trend is a gradual decrease in the activity of free functional groups due to proximity to groups occupied with metal ions [7,45]. The strongest pH drop occurred in systems with initial pH 6 and 7 and the weakest one for systems with pH 3. However, the number of released protons (calculated as value 10 raised to power (–pH)) at the highest Zn(II) concentration revealed that the vast majority of H^+^ ions were released to the solution at pH 3 while the lowest number at pH 7. The amount of zinc bound by 1 g of FAs could be calculated assuming the interaction of metal with the active sites of FAs at a ratio of 1:2. These results are shown in Figure 8 as a function of pH.

It is not surprising that the highest number of protonated COOH groups was present in pH 3, enabling interaction with a large amount of Zn ions by exchange with protons. However, an interesting fact is the release of a higher number of H^+^ at pH 5 than at pH 4. Probably, a new population of COOH groups become available and visible at pH 5 due to the reconfiguration and spatial development of the structure [80]. Zn(II) binding on the way of proton exchange at pH 7 was very low, which was associated with almost complete dissociation of the functional groups at these conditions [81]. The process of proton exchange was the strongest for FA1, which was the sample with the highest number of COOH groups, and for FA2, which exhibited the highest humification (Q4/6) and aromaticity degree (Q280). This indicated the importance of these chemical properties in the context of Zn(II) binding.

### 2.7. The Importance of the FA-Zn(II) Interaction Mechanism for Environment

The results of the study seem to be important due to fact that Zn is an essential micronutrient necessary for the proper growth and development of plants. So far, numerous studies have revealed that the best digestible forms of Zn are complex compounds with soil organic molecules [82,83,84]. The determined parameters describing the process of Zn binding by FAs can be helpful in optimizing fertilization in the event of deficit of this element. Understanding the relationship between the parameters describing the process of interaction and the chemical properties of FAs makes it possible to predict the direction, intensity of Zn(II) binding and stability of this sorption in the environment. For example, Brahmia et al. [85] reported that interactions between humic substances and napropamide depended on the elemental composition of humic substances: the binding increased with aromaticity and the N/C atomic ratio whereas it decreased with the elevation of O/C. In our studies, we verified the existence of similar relationships for FAs and Zn(II) using a simple correlation analysis and the t-student test. In this case however, the results did not reveal any significant relationship between the parameters describing Zn binding (*f_a_*, log*K_a_*, n, Zn_H+_/1g FA) and FAs properties (H/C, O/C, O/H, ω, COOH, OH, Q280, Q2/Q6, Q4/Q6), which suggests a complex nature of interaction with the metal—independent of only one feature of FA.

The only statistically significant relationships were found between some parameters determined for the complexation process (fluorescence studies) and those describing proton exchange of the functional groups (potentiometric titration) (Table 4). In the analysis above, the fraction of accessible fluorophores, *f_a_*, increased with Zn(II) binding during the exchange with protons at pH 7. This result, however, should be approached as inconclusive, and it requires further evaluation because a significant part of the functional groups (especially COOH) is dissociated at pH 7, and exchange in the Zn/H^+^ system plays a less important role. In turn, it should be mentioned that the literature on Zn binding by another fraction of humic substances, namely humic acids (more aromatic, with a higher molecular weight) suggest a relationship between the parameters describing Zn binding and some humic acids properties, which could be used for environmental forecasts. For example, negative correlations were revealed for stability constants of humic acids-Zn(II) complexes at pH 5 and 7 and Q4/Q6 and ∆logK (humification degree) [27]. A relationship between COOH + OH and complexation capacity was also reported for compounds formed by humic acids from soil and pig slurry with Zn(II) and Cu(II) ions [86]. In the case of FA fraction, our studies demonstrated the existence of various interaction mechanisms which are likely to reduce the effect of FA properties on the parameter describing a particular mechanism. The lack of a statistically significant effect of a given FA chemical property on the Zn binding process may also result from a generally lower stability of FA-Zn(II) compounds as compared to humic acids-Zn(II) systems. Lower stability constants for FA complexes are reported also in other studies [12,36,65]. Such a condition occurs in FAs in spite of them having an abundance of acidic functional groups compared to humic acids. We suppose that the structure of humic acids, containing of a greater number of aromatic components, is conducive to creating chelate compounds of considerable stability as compared to FAs containing a higher percentage of COOH groups but a lower number of aromatic rings.

Zn(II) also forms compounds of a generally lower stability than that of FAs with other metals [87]. It may be explained by the 3d10 configuration of Zn with completely filled d orbitals. The relatively low stability of FA-Zn(II) complexes can be a significant agricultural problem due to the frequent deficit of bioavailable forms of this element for plants. Therefore, research on determining conditions of the highest stability of the complexes may be of key importance for metal retention in soil and improvement of its absorption by the plant. Our results indicated that the stability of FA-Zn(II) complex compounds as well as the efficiency of Zn(II) binding by FAs can be enhanced by increasing the pH of the solution to the 6–7 levels. A further increase of alkalinity might not be effective due to the increasing amount of Zn hydroxide forms of less agricultural importance. A similar trend of increasing stability of Zn compounds along with an increase in the pH was also observed for interactions with natural organic matter from surface waters [22]. On the other hand, at low pH, Zn(II) complexing processes are of lower importance, and the metal occurs mainly in free form, the functional groups of organic molecules are slightly dissociated, and interactions with FAs are not as stable. Zn in these conditions is quite mobile and can undergo rapid leaching from arable soil layer.

## 3. Materials and Methods

### 3.1. Fulvic Acids

FAs were isolated from A-horizons of five chemically different soils: Haplic Fluvisol, Haplic Chernozem, Mollic Gleysol, Haplic Cambisol and Stagnic Luvisol using the procedure recommended by the International Humic Substances Society (IHSS) [88]. The degree of sample purity was evaluated by the analysis of ash content (A). In order to characterize the structure of FAs, the atomic ratios of H/C, O/C and O/H were determined using the CHNS/O 2400 analyzer (Perkin Elmer, Waltham, MA, USA). The internal oxidation degree (ω) was calculated with the Zdanov equation ω = [(2O + 3N) − H]/C [27]. The parameters of Q465/Q668 and Q254/Q436 expressing the humification degree and content of the compounds resistant to humification were calculated as a ratio of absorbance of aqueous FAs solution (40 mg dm^−3^) in 0.05 M NaHCO_3_, respectively at 465 and 668 nm, as well as at 254 and 436 nm, using an UV-Vis spectrometer (Jasco V-520, Tokyo, Japan). The value of absorbance recorded at 280 nm was used to evaluate structure aromaticity. Three replications were performed for each measurement, and the obtained results were averaged. The content of carboxylic (COOH) and phenolic (OH) groups was determined and described earlier using the method proposed by Dragunowa and Kucharenko [35].

### 3.2. Fluorescence Measurements

Stock aqueous solution was prepared for each sample of FA at a concentration of 50 mg dm^−3^. The Zn(II) stock solution of 1000 mg dm^−3^ was prepared by dissolving ZnCl_2_⋅2H_2_O in deionized water. Then, for each FA, five series of solutions were prepared with Zn concentration from 0 to 50 mg dm^−3^ and the final FA concentration of 40 mg dm^−3^. The pH of the series was adjusted to 3, 4, 5, 6 and 7 ± 0.1 using diluted solutions of HCl or NaOH. The solutions were equilibrated under N_2_ atmosphere for 24 h. Afterwards, the pH was readjusted as needed, and all the series were analyzed for fluorescence signals. The fluorescent spectra were recorded as 3D excitation-emission matrices (EEM) with a scan speed of 12,000 nm min^−1^ using the Hitachi F-7000 FL luminescence spectrometer (Hitachi, Tokyo, Japan). The emission wavelength was scanned from 300 to 600 nm, whereas the excitation wavelength was raised sequentially by 5 nm steps in the range of 250–500 nm. The analyses were preceded by fluorescence calibration using quinine sulphate at λ_ex_ = 350 nm and λ_em_ = 450 nm. Spectral correction of the instrument was performed using rhodamine B [34,35]. The EEM data were processed into contour maps using the Surfer software (Golden Software Inc., Golden, CO, USA). The binding parameters were determined on the basis of changes in intensities of fluorescence peaks using fluorescence models and equations.

### 3.3. FTIR Measurements

Five series of solutions for each FA (40 mg dm^−3^) were prepared with selected Zn concentrations (0, 2, 5, 10, 50 mg dm^−3^) at pH 3, 4, 5, 6 and 7. The solutions were lyophilized. Then, 1 mg FA-Zn preparations were homogenized with 200 mg KBr of spectral purity and analyzed on a FTIR spectrometer (Tensor 27, Bruker, Billerica, MA, USA) in the range of 400–4000 cm^−1^. The characteristics were obtained as an average of three measurements with 256 scans at 2 cm^−1^ resolution each.

### 3.4. Metal-Proton Exchange Equilibria

The interaction of Zn(II) ions with FAs by proton exchange was analyzed using the titration method. Portions of Zn solution (1000 mg dm^−3^) with a fixed pH (3, 4, 5, 6 and 7) were added to FA solutions (40mg dm^−3^) of the corresponding pH (3, 4, 5, 6 and 7) to obtain the final Zn concentration from 0 to 50 mg dm^−3^. The solutions were stirred at a constant stirring speed under N_2_ atmosphere. The pH value was recorded after each titration step when the signal from the electrode was stable at the 0.001 level. The processing data related to the number of released protons enabled the evaluation of binding on the way of H^+^-Zn exchange. The measurements were performed in triplicate for each FA and each variant of pH and averaged afterwards.

### 3.5. Statistical Analysis

The relationships between the different mechanisms of Zn(II) binding were investigated using a correlation analysis for parameters describing interactions with Zn ions, i.e., stability constants, the number of binding sites and the fraction of accessible fluorophores (fluorescence spectroscopy method), as well as for parameters obtained from proton binding tests (potentiometric titration methods). The results were presented as a correlation matrix. Statistical significance was analyzed at α = 0.05 by using the t-student test. Normal data distribution was verified using the Saphiro–Wilk test.

## 4. Conclusions

The studies discussed above involved an effort of employing complementary analyses to molecular interactions between an important micronutrient—zinc, and the most mobile fraction of humic substances—fulvic acids. This article attempts to assess the impact of a wide range of pH (3–7), metal concentrations (0–50 mg dm^−3^), as well as the different origin and chemical properties of FAs on the mechanism, stability and efficiency of Zn binding.

The results obtained from the fluorescence, FTIR and potentiometric studies demonstrated that the key factor determining the type and efficiency of interactions was the pH and concentration of Zn(II) ions. The complexation process proved highly relevant at pH 6 and 7, whereas protons exchange prevailed at pH 3. Stability constant of the formed complexes increased along with the pH and exhibited the highest stability at pH7 (logK increase from ~3.8 to 4.2). Complexation was preferred by less-humidified structures of lower molecular mass containing more oxygen groups. The number of fluorophores available for Zn(II) increased from pH 3 to 7 by ~44%, however, the formation of hydrolyzed Zn species decreased the number of the binding sites, thus making the interactions less effective. FTIR spectra revealed that the bidentate chelate mode can be typical for low pHs. On the other hand, the monodentate mode at low Zn concentration and bidentate bridging at higher Zn(II) doses can dominate for higher pHs. The binding of Zn(II) ions revealed the highest dynamics at low metal concentration (below 10 mg dm^−3^) due to the high number of uncomplexed binding sites. However, ligands complexation was not completed even at excess Zn(II) due to the steric effect of the structures. The higher pH had a positive effect on stability of chemical bonds due to dissociation of subsequent functional groups, which caused greater attraction of Zn(II) cations. Mutual repulsion of charged structures counteracted destabilization of the soluble FA-Zn(II) complex and protected it from precipitation. Simultaneously, conformation of FA structure showed favorable changes from aggregated and coiled shape at low pHs to spatially developed and more linear structure at high pHs where Zn(II) access was easier.

Evaluation of the different models showed that Zn(II) binding was poorly modeled by the classic Stern–Volmer equation, and it was well described by the modified Stern–Volmer formula, assuming the existence of available and unavailable fluorophore populations (*R* > 0.98), as well as by a double logarithmic equation (*R* > 0.94). The ratio of fluorescence intensities of different fluorophores was proposed as an indicator of binding affinity of various structures.

The structural and sorption properties of FAs (H/C, O/C, O/H, COOH, OH, Q465/Q665, Q254/Q436) did not reveal any statistically significant effect on the intensity and mechanism of interaction with Zn(II). It confirms the complex nature of this process and a probable impact of many FAs properties. A positive relationship was discovered between the fraction of accessible fluorophores and Zn(II) binding at pH7 determined based on proton release (*R* = 0.91–0.97). Such a comprehensive analysis enables a better understanding and optimization of the interaction mechanism. We believe that the obtained results can be useful in controlling Zn mobility and bioavailability in different conditions, both in the event of its deficiency and toxic concentration levels.

## Figures and Tables

**Table 1:** Chemical and structural properties of soil fulvic acids (FAs).

FA No	FA Origin		H/C	O/C	O/H	ω	COOH	OH	Q465/Q665	Q254/Q436	Q280	A
	Soil type	Soil location	d.u.				(cmol kg^−1^)		d.u.			(%)
FA1	Haplic Fluvisol (Alluvial soil)	51°09′N/22°59′E	0.90	0.77	0.86	0.78	690	179	10.46	18.02	0.67	0.90
FA2	Haplic Chernozem (Chernozem)	50°32′N/24°01′E	0.94	0.97	1.04	1.12	628	173	9.07	14.90	0.60	0.83
FA3	Mollic Gleysol (Black Earth)	50°22′N/23°39′E	0.85	0.65	0.76	0.61	561	224	13.89	13.45	0.69	0.73
FA4	Haplic Cambisol (Brown Soil)	51°23′N/22°35′E	0.87	0.69	0.79	0.59	649	290	16.15	15.62	0.65	0.63
FA5	Stagnic Luvisol (Grey-brown soil)	50°38′N/22°41′E	0.91	0.73	0.80	0.66	632	229	10.36	12.53	0.64	1.63

ω—internal oxidation degree, Qx/Qy—ratio of absorbances at particular wavelength, A—ash content, d.u.—dimensionless unit.

**Table 2:** Maximum values of fluorescence intensities of FAs regarding A and B peaks at pH 3–7.

	pH 3		pH 4		pH 5		pH 6		pH 7	
	FI Peak A	FI Peak B	FI Peak A	FI Peak B	FI Peak A	FI Peak B	FI Peak A	FI Peak B	FI Peak A	FI Peak B
FA1	893	757	936	812	1043	911	1044	942	1085	973
FA2	817	681	801	690	873	780	895	818	898	834
FA3	434	430	453	455	515	523	532	555	534	565
FA4	599	516	717	627	852	713	838	712	845	732
FA5	559	469	635	547	760	622	728	615	661	568

**Table 3:** Binding parameters calculated from modified Stern–Volmer equation and double logarithmic equation for FA complexes with Zn(II).

	**FA1**											
	**A-peak**						**B-peak**					
	***f_a_***	**log*K_a_***	**R**	**log*K_b_***	**n**	**R**	***f_a_***	**log*K_a_***	**R**	**log*K_b_***	**n**	**R**
pH 3	n.a.	n.a.	n.a.	n.a.	n.a.	n.a.	n.a.	n.a.	n.a.	n.a.	n.a.	n.a.
pH 4	n.a.	n.a.	n.a.	n.a.	n.a.	n.a.	n.a.	n.a.	n.a.	0.98	0.54	0.971
pH 5	0.28	3.86	0.990	1.47	0.61	0.993	0.32	3.87	0.991	1.56	0.62	0.992
pH 6	0.46	3.99	0.997	1.68	0.62	0.981	0.52	4.09	0.997	1.72	0.61	0.980
pH 7	0.58	4.11	0.997	1.74	0.60	0.962	0.64	4.23	0.997	1.77	0.57	0.960
	**FA2**											
	**A-peak**						**B-peak**					
	***f_a_***	**log*K_a_***	**R**	**log*K_b_***	**n**	**R**	***f_a_***	**log*K_a_***	**R**	**log*K_b_***	**n**	**R**
pH 3	n.a.	n.a.	n.a.	n.a.	n.a.	n.a.	n.a.	n.a.	n.a.	n.a.	n.a.	n.a.
pH 4	n.a.	n.a.	n.a.	n.a.	n.a.	n.a.	n.a.	n.a.	n.a.	n.a.	n.a.	n.a.
pH 5	0.27	3.81	0.989	1.41	0.65	0.988	0.28	3.95	0.984	1.40	0.61	0.995
pH 6	0.45	4.05	0.995	1.57	0.58	0.980	0.49	4.18	0.992	1.54	0.53	0.984
pH 7	0.55	4.18	0.998	1.62	0.55	0.970	0.59	4.35	0.995	1.58	0.50	0.977
	**FA3**											
	**A-peak**						**B-peak**					
	***f_a_***	**log*K_a_***	**R**	**log*K_b_***	**n**	**R**	***f_a_***	**log*K_a_***	**R**	**log*K_b_***	**n**	**R**
pH 3	n.a.	n.a.	n.a.	n.a.	n.a.	n.a.	n.a.	n.a.	n.a.	n.a.	n.a.	n.a.
pH 4	n.a.	n.a.	n.a.	n.a.	n.a.	n.a.	n.a.	n.a.	n.a.	n.a.	n.a.	n.a.
pH 5	0.31	3.85	0.995	1.49	0.63	0.986	0.33	3.99	0.995	1.36	0.56	0.984
pH 6	0.45	4.11	0.993	1.43	0.55	0.970	0.50	4.18	0.996	1.50	0.54	0.971
pH 7	0.52	4.21	0.996	1.45	0.55	0.959	0.59	4.28	0.996	1.53	0.54	0.951
	**FA4**											
	**A-peak**						**B-peak**					
	***f_a_***	**log*K_a_***	**R**	**log*K_b_***	**n**	**R**	***f_a_***	**log*K_a_***	**R**	**log*K_b_***	**n**	**R**
pH 3	n.a.	n.a.	n.a.	n.a.	n.a.	n.a.	n.a.	n.a.	n.a.	n.a.	n.a.	n.a.
pH 4	n.a.	n.a.	n.a.	n.a.	n.a.	n.a.	n.a.	n.a.	n.a.	n.a.	n.a.	n.a.
pH 5	0.23	3.88	0.976	1.43	0.66	0.992	0.28	3.85	0.984	1.42	0.65	0.985
pH 6	0.44	3.92	0.996	1.85	0.61	0.969	0.51	3.90	0.985	1.72	0.64	0.952
pH 7	0.50	4.13	0.993	1.58	0.59	0.974	0.54	4.21	0.993	1.56	0.55	0.969
	**FA5**											
	**A-peak**						**B-peak**					
	***f_a_***	**log*K_a_***	**R**	**log*K_b_***	**n**	**R**	***f_a_***	**log*K_a_***	**R**	**log*K_b_***	**n**	**R**
pH 3	0.02	3.99	0.941	n.a.	n.a.	n.a.	n.a.	n.a.	n.a.	n.a.	n.a.	n.a.
pH 4	0.05	3.83	0.890	0.88	0.69	0.954	n.a.	n.a.	n.a.	1.03	0.67	0.937
pH 5	0.22	3.86	0.977	1.39	0.64	0.997	0.26	3.80	0.987	1.49	0.63	0.993
pH 6	0.47	3.68	0.997	1.88	0.86	0.976	0.48	3.79	0.998	1.84	0.81	0.973
pH 7	0.49	3.92	0.993	1.75	0.70	0.974	0.52	4.09	0.996	1.67	0.61	0.976

n.a. equation not applicable.

**Table 4:** Coefficients of Person’s correlation calculated between fraction of accessible fluorophores, *f_a_*, stability constants, log*K_a_*, number of binding sites, *n*, (determined from fluorescence spectroscopy) and Zn(II) binding efficiency (determined from proton releasing studies) at pH 5, 6 and 7. Bold digits highlight statistically significant relationships for α = 0.05.

	N_Zn_/g FA pH 5		N_Zn_/g FA pH 6		N_Zn_/g FA pH 7
*f_a_*A pH 5	0.84	*f_a_*A pH 6	0.05	*f_a_*A pH 7	0.91
*f_a_*B pH 5	0.80	*f_a_*B pH 6	0.39	*f_a_*B pH 7	0.97
log*K_a_*A pH 5	−0.58	log*K_a_*A pH 6	0.73	log*K_a_*A pH 7	0.66
log*K_a_*B pH 5	0.65	log*K_a_*B pH 6	0.86	log*K_a_*B pH 7	0.76
nA pH 5	−0.47	nA pH 6	−0.60	nA pH 7	−0.65
nB pH 5	−0.49	nB pH 6	−0.73	nB pH 7	−0.56
